# Examining the Influence of Cognitive Load and Environmental Conditions on Autonomic Nervous System Response in Military Aircrew: A Hypoxia–Normoxia Study

**DOI:** 10.3390/biology13050343

**Published:** 2024-05-14

**Authors:** Harrison L. Wittels, S. Howard Wittels, Michael J. Wishon, Jonathan Vogl, Paul St. Onge, Samantha M. McDonald, Leonard A. Temme

**Affiliations:** 1 Tiger Tech Solutions, Inc., Miami, FL 33140, USA; hl@tigertech.solutions (H.L.W.); shwittels@gmail.com (S.H.W.); joe@tigertech.solutions (M.J.W.); 2 Department of Anesthesiology, Mount Sinai Medical Center, Miami, FL 33140, USA; 3 Department of Anesthesiology, Wertheim School of Medicine, Florida International University, Miami, FL 33199, USA; 4 Miami Beach Anesthesiology Associates, Miami, FL 33140, USA; 5 Army Aeromedical Research Laboratory, Fort Novosel, AL 36362, USA; jonathan.f.vogl.civ@health.mil (J.V.); paul.m.stonge2.civ@health.mil (P.S.O.); leonard.a.temme.civ@health.mil (L.A.T.); 6 School of Kinesiology and Recreation, Illinois State University, Normal, IL 61761, USA

**Keywords:** cognitive load, military, hypoxia, normoxia, autonomic nervous system, sympathetic drive, aviators

## Abstract

**Simple Summary:**

During combat missions, military aircrew rely on their ability to make accurate life-or-death decisions within infinitesimal timeframes. Research shows that performing more challenging tasks (e.g., high cognitive load) requires a greater supply of oxygen to the brain, a process regulated by the autonomic nervous system. However, the influence of prolonged exposure to performing low and high cognitive loads, specifically related to flight operations, is unclear. Thus, the current study evaluated the response of the autonomic nervous system to sustained performance of tasks requiring low or high cognitive functions under two different environmental conditions: hypoxia (i.e., reduced oxygen content) and normoxia (i.e., standard oxygen content). Twenty military and civilian adults (19 to 45 years old) participated in the study, and each performed a series of low- and high-cognitive-load assessments for nearly two hours. All subjects performed these tests separately under hypoxic (14.0% oxygen) and normoxic (21.0% oxygen) conditions. Autonomic nervous system function was measured using a monitoring device placed on the upper right arm that collected heart rate and heart rate variability data. Our study found that prolonged exposure to high cognitive loads induced heightened activity of the autonomic nervous system, with subjects eliciting higher heart rates and lower heart rate variability relative to low cognitive loads. Additionally, the influence of high cognitive load was greater in the hypoxic environment. Accounting for the response of the autonomic nervous system to cognitive loads is important, as this stress likely compounds to the existing fight-or-flight response occurring during pre-flight and in-flight operations.

**Abstract:**

Executing flight operations demand that military personnel continuously perform tasks that utilize low- and high-order cognitive functions. The autonomic nervous system (ANS) is crucial for regulating the supply of oxygen (O2) to the brain, but it is unclear how sustained cognitive loads of different complexities may affect this regulation. Therefore, in the current study, ANS responses to low and high cognitive loads in hypoxic and normoxic conditions were evaluated. The present analysis used data from a previously conducted, two-factor experimental design. Healthy subjects (*n* = 24) aged 19 to 45 years and located near Fort Novosel, AL, participated in the parent study. Over two, 2-h trials, subjects were exposed to hypoxic (14.0% O_2_) and normoxic (21.0% O_2_) air while simultaneously performing one, 15-min and one, 10-min simulation incorporating low- and high-cognitive aviation-related tasks, respectively. The tests were alternated across five, 27-min epochs; however, only epochs 2 through 4 were used in the analyses. Heart rate (HR), HR variability (HRV), and arterial O_2_ saturation were continuously measured using the Warfighter Monitor^TM^ (Tiger Tech Solutions, Inc., Miami, FL, USA), a previously validated armband device equipped with electrocardiographic and pulse oximetry capabilities. Analysis of variance (ANOVA) regression models were performed to compare ANS responses between the low- and high-cognitive-load assessments under hypoxic and normoxic conditions. Pairwise comparisons corrected for familywise error were performed using Tukey’s test within and between high and low cognitive loads under each environmental condition. Across epochs 2 through 4, in both the hypoxic condition and the normoxic condition, the high-cognitive-load assessment (MATB-II) elicited heightened ANS activity, reflected by increased HR (+2.4 ± 6.9 bpm) and decreased HRV (−rMSSD: −0.4 ± 2.7 ms and SDNN: −13.6 ± 14.6 ms). Conversely, low cognitive load (ADVT) induced an improvement in ANS activity, with reduced HR (−2.6 ± 6.3 bpm) and increased HRV (rMSSD: +1.8 ± 6.0 ms and SDNN: vs. +0.7 ± 6.3 ms). Similar observations were found for the normoxic condition, albeit to a lower degree. These within-group ANS responses were significantly different between high and low cognitive loads (HR: +5.0 bpm, 95% CI: 2.1, 7.9, *p* < 0.0001; rMSSD: −2.2 ms, 95% CI: −4.2, −0.2, *p* = 0.03; SDNN: −14.3 ms, 95% CI: −18.4, −10.1, *p* < 0.0001) under the hypoxic condition. For normoxia, significant differences in ANS response were only observed for HR (+4.3 bpm, 95% CI: 1.2, 7.4, *p* = 0.002). Lastly, only high cognitive loads elicited significant differences between hypoxic and normoxic conditions but just for SDNN (−13.3 ms, 95% CI, −17.5, −8.9, *p* < 0.0001). Our study observations suggest that compared to low cognitive loads, performing high-cognitive-load tasks significantly alters ANS activity, especially under hypoxic conditions. Accounting for this response is critical, as military personnel during flight operations sustain exposure to high cognitive loads of unpredictable duration and frequency. Additionally, this is likely compounded by the increased ANS activity consequent to pre-flight activities and anticipation of combat-related outcomes.

## 1. Introduction

Combat missions impose prolonged exposure to cognitive loads upon military personnel, especially aircrew [1,2]. Effectively executing aviation-related tasks utilizing low- or high-order cognitive function requires a sufficient supply of oxygen (O_2_) to the brain [3,4,5]. Importantly, the autonomic nervous system (ANS) regulates the physiological processes involved in cerebral O_2_ supply, processes like arterial vasodilation, cardiac output, ventilation, etc. [6]. Thus, preserving ANS function and, subsequently, the integrity of cognitive function is paramount in preventing catastrophic events, especially during missions performed in adverse environments.

In-flight operations expose aircrew to altitude levels that compromise ANS function and cognitive performance [7,8]. Studies demonstrating these negative effects were largely conducted under severe hypoxic conditions (>10% O_2_) [9,10], yet rotary-wing aircrew spend more time flying at lower altitudes (8000 to 10,000 ft), which induce a mildly hypoxic (13 to 16% O_2_) environment. The effects of mild hypoxia on ANS function, however, are understudied, especially for evaluating the simultaneous exposure to cognitive load. Only one study, to our knowledge, evaluated the dual exposure of mild hypoxia and sustained performance of simulated aviation-related tasks and found significantly altered ANS function among aircrew [11]. Specifically, in that study, subjects elicited increased heart rates (HR), reduced HR variability (HRV), and decreased O_2_ saturation [11]. What remains less clear, however, is whether ANS function is differentially affected by the complexity of cognitive load under mild hypoxia. Aircrew engage in a wide spectrum of tasks like surveillance, air-to-air refueling, transport of hazard materials, and search and rescue, which utilize both low- and high-order cognitive functions like visual processing, memory recall, information integration, and decision-making [12,13].

Quantifying the potential differential effects of aviation-related tasks of varying complexity on the ANS response to mild hypoxia is critical to the health and safety of military aircrew. This information may allow military personnel to identify effective strategies to prevent ANS deterioration, thereby optimizing cognitive performance during missions and protecting the lives of aircrew. The purpose of the current study was to evaluate the responses of the ANS to low- and high-cognitive-load assessments performed under hypoxic and normoxic conditions. We hypothesize that (1) performing higher-cognitive-load tasks would elicit a greater ANS response, reflected by higher HR and lower HRV relative to low cognitive tasks, and (2) the observed ANS responses would be exaggerated under the hypoxic condition compared to the normoxic condition.

## 2. Materials and Methods

### 2.1. Study Design and Sample

The current study performed post hoc analyses utilizing data from a previously conducted study that employed a mixed-model, within-subject, two-factor (A and B) experimental design. In the parent study, all subjects (*n* = 24) were sequentially exposed to two different levels of O_2_ (Factor A). Factor A condition 1 required subjects to continuously breathe normobaric–hypoxic air (14.0% O_2_) for 108 min and then, for 26 min, continuously breathe normobaric–normoxic air (21.0% O_2_). Factor A condition 2 required subjects to continuously breathe normobaric–normoxic air (21.0% O_2_) for 108 min and then, breathe normobaric–hyperoxic air (33.0% O_2_) for 26 min. Subjects completed both trials in one day, with one occurring in the morning and the other in the afternoon following a 60 min lunch break. The order of the scheduled testing alternated between subjects to counterbalance the data. Factor B was cognitive loads imposed upon subjects that required nearly continuous performance of simulated flight-relevant tasks of varying complexity. The tasks were performed for a total of 134 min during each condition of Factor A.

### 2.2. Subjects

Twenty-four subjects from the local geographical area around Fort Novosel, AL, USA, participated in the parent study and consisted of both military and civilian personnel. Subjects were eligible for the study if they were (1) between 19 years and 45 years of age, (2) in an “off-duty” status, (3) not pregnant, and (4) reported no history of corrective eye muscle surgery, strabismus, amblyopia, or severe altitude-related sickness. Exclusion criteria for the parent study included (1) presenting symptoms of respiratory or sinus infection or flu, (2) having used tobacco products within the 6 months preceding the study, (3) having donated blood within 30 days of the trial, (4) having spent ≥ 10 days at an altitude >5000 ft above mean sea level in the last 3 months, (5) having a history of excessive alcohol use within the past 6 months, and (6) receiving medical treatment for anemia, asthma, cardiovascular disease, hypertension, sick cell anemia, emphysema, seizure disorder, chronic stress, concussion with loss of consciousness, attention-deficit hyperactivity disorder, or psychiatric, neurological, or sleep-related conditions. Prior to the onset of the parent study, all subjects were fully informed of the study protocol, risks, benefits, and voluntariness of their participation in accordance with the Declaration of Helsinki. Following, all eligible subjects voluntarily consented to participate in the parent study. By agreement with the subjects, the gender and ethical attributes of the subjects cannot be disclosed. All aspects of the parent study were approved by the Headquarters, U.S. Army Medical Research and Development Command Institutional Review Board (HQ USAMRDC IRB M-10859, approval date 28 December 2020).

### 2.3. O_2_ Exposure (Factor A)

The three pre-mixed compressed air gas combinations were obtained from a commercial supplier (Airgas, Air Liquide Company, Radnor Township, Philadelphia, PA, USA). These pre-mixed combinations were (a) 21.0% O_2_ and 79.0% N to approximate the atmospheric air at mean sea level (MSL), (b) 14.0% O_2_ and 86.0% N to approximate the O_2_ content of the atmospheric air typically encountered around 10,000 feet above MSL, and (c) 33.0% O_2_ and 67.0% N to provide a moderately O_2_-enriched gas. Subjects breathed these gases via a standard aviator mask breathing unit typically used by high-performance fighter-jet pilots (MBU-20/P, Gentex Corp., Zeeland, MI, USA). The mask was fitted to comfortably and securely seal over the oronasal cavity with an appropriately sized aviator helmet. The delivery of the gases to the mask occurred via a flexible hose attached to the outlet of a standard O_2_ regulator designed for use in high-performance aircraft (Cobham 29,270, CRU-73 automatic diluter-demand pressure-breathing O_2_ regulator). The inlet supply pressure was set at 50 psig (344.7 kPa) and regulated by the built-in pressure reducer.

### 2.4. Cognitive Load: Simulated In-Flight Tasks (Factor B)

**Figure 1:**
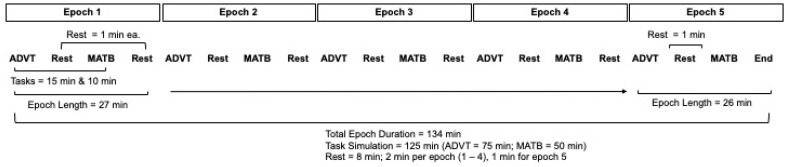
Schematic of the parent study trial design.

The cognitive loads performed by the subjects under the conditions of Factor A consisted of two aviation-relevant computer-simulated task batteries: Automated Desktop Vision Tester (ADVT) [14] and Enhanced Air Force Multi-Attribute Task Battery (EAF-MATB-II) [15,16]. The tasks were alternated five times, equating to 125 min of continuous performance testing. A one-minute period was provided for study personnel to switch between the two sets of task batteries, resulting in a total trial time of 134 min (see Figure 1). The 134 min trial was divided into 5 separate epochs, each lasting 27 min, with 25 min allocated to exposing subjects to alternating cognitive loads and two one-minute “rest” periods to transition from one task to the other. The 5th epoch did not include the final 1 min transition since the trial had terminated. For the purposes of the current study, however, the timeframes during which the subjects were initially exposed to different conditions of Factor A: normoxic to hypoxic (epoch 1), normoxic to normoxic (epoch 1), hypoxic to normoxic (epoch 5), and normoxic to hyperoxic (epoch 5) were not included in the analyses. During epoch 1, subjects were simultaneously exposed to Factors A and B. As such, the independent effects of cognitive load could not be teased out. Epoch 5 did not align with the focus of the current study.

#### Automated Desktop Vision Tester and Enhanced Air Force Multi-Attribute Task Battery

The ADVT was 15 min in duration and consisted of a set of simulated vision tests measuring conventional static stereo acuity, dynamic stereo acuity, and two-dimensional tracking, in addition to horizontal and vertical fusion range determinations [14]. The specific ADVT tests administered include the following: Conventional Stereo Acuity Near Threshold (CSA N.T.), Conventional Stereo Acuity Far Threshold (CSA F.T.), Dynamic Stereo Acuity (D.S.A.), 2D Positional Tracking (2D P.T.), and Fusional Range (F.R.). The EAF-MATB testing lasted 10 min and consisted of several simulated aviation tasks, including visual target tracking, auditory and visual signal monitoring, resource management, and responses to event onsets. The task complexity of the ADVT and EAF-MATB-II tests varied, with each differentially categorized as low and high cognitive load, respectively.

### 2.5. Autonomic Nervous System Response (Outcome)

HR and HRV measures are strong indicators of ANS activity and therefore represented the ANS response to hypoxia, normoxia, and cognitive load. HR is defined as the number of cardiac contractions occurring during a 60 s interval (beats per min; bpm). HRV is defined as the time variation between heartbeats, and the metrics used included SDNN: standard deviation of NN intervals and rMSSD: the root mean square of successive differences, which are described in detail elsewhere [17,18]. HR and HRV were continuously measured throughout each experimental trial except during the 60 min break between trials. Pulse oximetry was also continuously measured (SpO_2_) to estimate the level of arterial O_2_ saturation in the blood [19,20]. These ANS responses were measured via an armband with electrocardiographic and pulse oximetry capabilities (Warfighter Monitor^TM^ (WFM), Tiger Tech Solutions, Inc., Miami, FL, USA), which were previously validated in diverse populations [21]. The WFM was fitted to the posterior aspect of the subjects’ upper right arm and secured with an elastic band. The WFM did not interfere with subjects’ movement patterns.

### 2.6. Statistical Analysis

Within- and between-group tests were performed using two-factor analysis of variance (ANOVA) models. These models evaluated the following: (1) differences in HR, rMSSD, SDNN, and SpO_2_ across epochs 1, 2, 3, and 4 separately for the ADVT and MATB-II, stratified by the Factor A conditions (hypoxic and normoxic); (2) differences in HR, rMSSD, SDNN, and SpO_2_ between the ADVT and EAF-MATB-II tests for epochs 1, 2, 3, and 4 within each Factor A condition (hypoxic and normoxic); and (3) differences in HR, rMSSD, SDNN, and SpO_2_ between Factor A conditions (hypoxic vs. normoxic) separately for the ADVT and EAF-MATB-II. Additionally, box-and-whisker plots were created to graphically represent the within- and between-group differences. All groups were tested for normality using Kolmogorov–Smirnov testing and satisfied the normal distribution assumption of linear regression. Statistical analysis was performed using MATLAB R2022a. Statistical significance was set a priori at <0.05.

## 3. Results

### 3.1. ANS Response to High and Low Cognitive Loads

The responses of the ANS during the ADVT and MATB-II tests across epochs 2 through 4 are presented in Table 1. For each test, the values represent the changes in ANS activity metrics from the start until the end of each test within a given epoch.

#### 3.1.1. Hypoxic Condition

Performing the MATB-II assessment significantly altered ANS activity. Specifically, in epoch 2, HR increased +1.8 bpm, with concomitant decreases in HRV (rMSSD: −0.4 ± 2.4 ms and SDNN: −13.8 ± 15.7 ms) and arterial O_2_ saturation (SpO_2_: −0.2 ± 0.1%). These same trends, mostly for HR and SDNN, were observed for epochs 3 and 4. When averaged across the three epochs, HR increased by 2.4 (6.9) bpm while HRV metrics decreased, with a large reduction shown for SDNN (−13.6 ± 14.7 ms). SpO_2_ changed minimally following the initial exposure to hypoxia in epoch 1, reflected by a 0.1 (0.3) % change. For the ADVT assessment, subjects elicited, on average, slight improvements in ANS function, decreases in HR (−2.6 ± 6.3 bpm) and increases in HRV (rMSSD: 0.72 ± 6.3 ms and SDNN: 1.8 ± 6.0 ms). Arterial O_2_ saturation exhibited negligible positive changes (SpO_2_: +0.4 to +0.79%).

#### 3.1.2. Normoxic Condition

Like the hypoxic condition, during the MATB-II assessment under normoxia, subjects exhibited, on average, an increase HR (+1.8 ± 6.4 bpm) and decreases in HRV for both rMSSD (−0.3 ± 2.8 ms) and SDNN (−0.3 ± 1.4 ms). SpO_2_ negligibly increased across epochs 2 through 4 (+0.1 ± 0.2%). For the ADVT assessment under the normoxic condition, the subjects’ HR appeared to decrease (−2.5 ± 3.9 bpm). Additionally, increases in HRV were observed for both rMSSD and SDNN (rMSSD: +1.0 ± 4.2 ms and SDNN: +1.7 ± 4.3 ms, respectively). Like the MATB-II, SpO_2_ minimally changed across epochs 2, 3, and 4 (+0.1 ± 0.1%).

### 3.2. Differences in ANS Response between High and Low Cognitive Loads

The differences in the ANS responses between the MATB-II and ADVT assessments throughout epochs 2 through 4 are presented in Table 2.

#### 3.2.1. Hypoxic Condition

Under hypoxia, both statistically significant and non-significant differences in the changes in ANS response between the MATB-II and ADVT assessments were observed across epochs 2, 3, and 4. Statistically significant differences were observed for HR, HRV, and arterial O_2_ saturation. Specifically, compared to the ADVT assessment, subjects performing the MATB-II demonstrated a greater change in HR (+5.0 bpm, 95% CI: 2.1, 7.9, *p* < 0.0001), HRV (rMSSD: −2.2 ms, 95% CI: −4.2, −0.2, *p* = 0.03, and SDNN: −14.3 ms, 95% CI: −18.4, −10.1, *p* < 0.0001), and arterial O_2_ saturation (SpO_2_: −0.3%, 95% CI: −0.4, −0.2, *p* < 0.0001). Within epochs 2, 3, and 4, SDNN appeared to be the only ANS response that consistently showed a significantly large change. Otherwise, the differences in the changes for HR, rMSSD, and SpO_2_ were mostly non-significant between the MATB-II and ADVT when analyzed within each epoch.

#### 3.2.2. Normoxic Condition

Similar observations were found under the normoxic condition, with heightened ANS activity presenting among subjects performing the MATB-II assessments. The largest difference observed between the MATB-II and ADVT was HR. When averaged across epochs 2, 3, and 4, a significant difference was found for HR (+4.3 bpm, 95% CI: −7.4, −1.2, *p* = 0.002). As expected under the normoxic condition, no significant differences in arterial O_2_ saturations were observed across epochs 1 through 4. Interestingly, however, changes in HRV indices between the MATB-II and ADVT assessments were not significantly different.

### 3.3. Differences in the ANS Response to Cognitive Load between Factor A Conditions

The differences in the change in ANS activity during the MATB-II and ADVT assessments between hypoxic and normoxic conditions are presented in Table 3.

#### 3.3.1. MATB-II

The changes in ANS activity occurring during the MATB-II assessments differed between hypoxic and normoxic conditions. The decrease in SDNN observed (see Table 1) while subjects performed the MATB-II was significantly larger in the hypoxic exposure relative to the normoxic exposure during epochs 2, 3, and 4 (−13.3 ms, 95% CI: −17.5, −8.9, *p* < 0.0001). Interestingly, no other significant differences were observed in the changes in ANS activity between the hypoxic and normoxic conditions during the MATB-II assessment.

#### 3.3.2. ADVT

**Figure 2:**
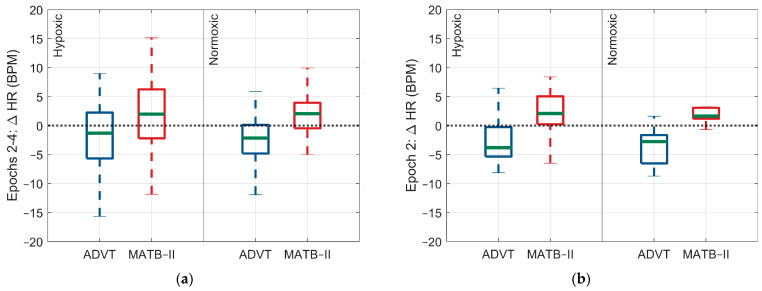

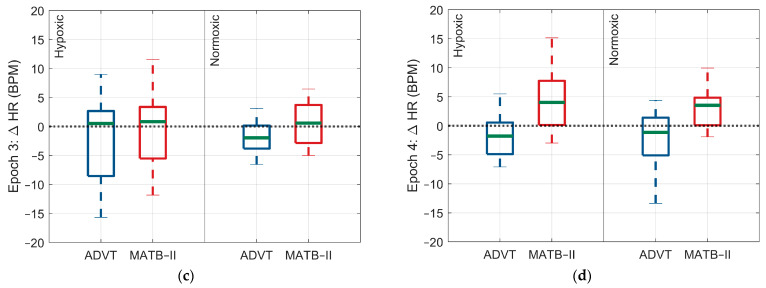
Differences in the HR response within and between low and high cognitive load assessments by epoch and within and between Factor A conditions. (**a**) Change in HR response across epochs 2 through 4, (**b**) change in HR response during epoch 2, (**c**) change in HR response during epoch 3, (**d**) change in HR response during epoch 4. The blue and red indicate each test administered, ADVT and MATB-II, respectively.

**Figure 3:**
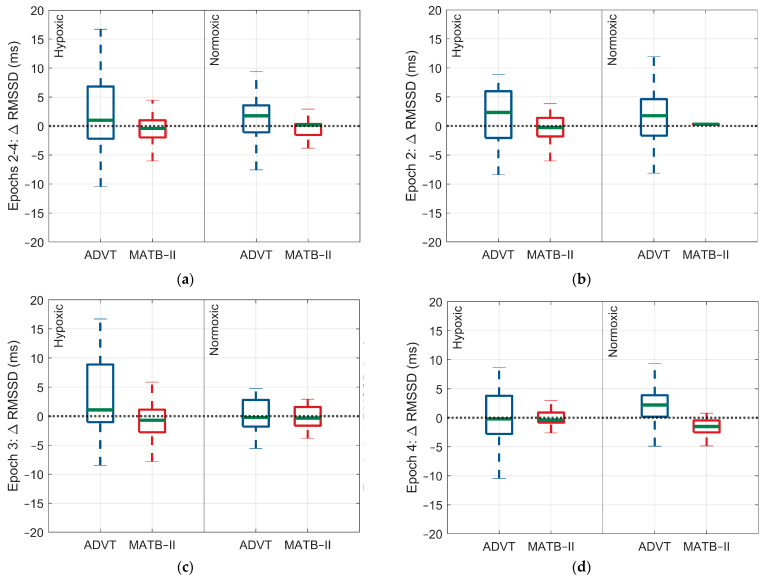
Differences in the rMSSD response within and between low and high cognitive load assessments by epoch and within and between Factor A conditions. (**a**) Change in rMSSD response across epochs 2 through 4, (**b**) change in rMSSD response during epoch 2, (**c**) change in rMSSD response during epoch 3, (**d**) change in rMSSD response during epoch 4. The blue and red indicate each test administered, ADVT and MATB-II, respectively.

**Figure 4:**
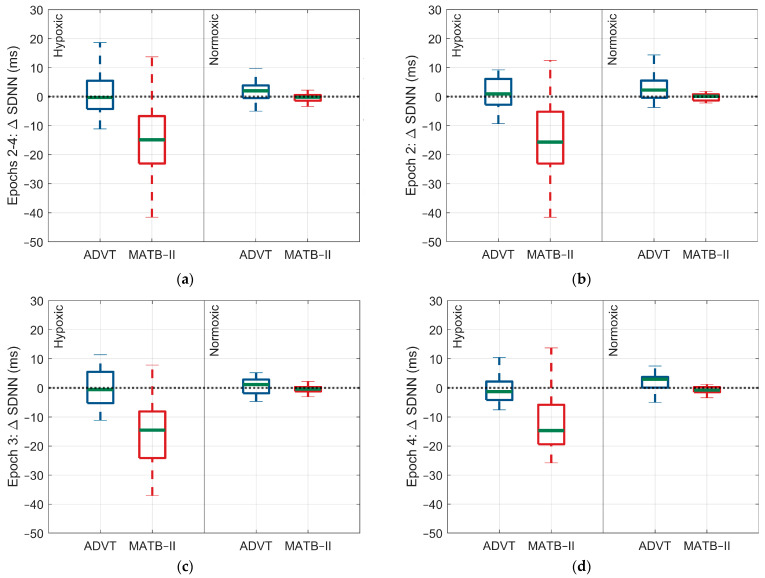
Differences in the SDNN response within and between low and high cognitive load assessments by epoch and within and between factor A conditions. (**a**) Change in SDNN response across epochs 2 through 4, (**b**) change in SDNN response during epoch 2, (**c**) change in SDNN response during epoch 3, (**d**) change in SDNN response during epoch 4. The blue and red indicate each test administered, ADVT and MATB-II, respectively.

**Figure 5:**
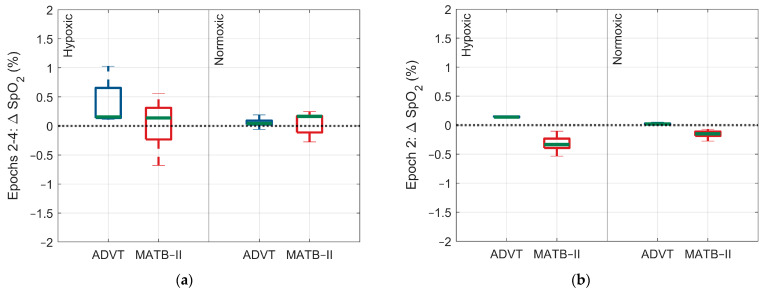

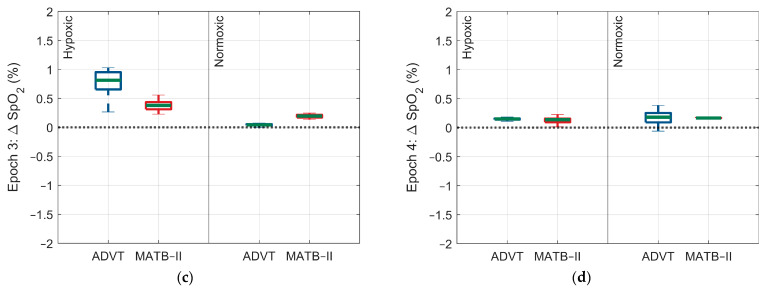
Differences in the SpO_2_ response within and between low and high cognitive load assessments by epoch and within and between Factor A conditions. (**a**) Change in SpO_2_ response across epochs 2 through 4, (**b**) change in SpO_2_ response during epoch 2, (**c**) change in SpO_2_ response during epoch 3, (**d**) change in SpO_2_ response during epoch 4. The blue and red indicate each test administered, ADVT and MATB-II, respectively.

For the ADVT assessment, there were no significant differences in ANS activity occurring between hypoxic and normoxic conditions. However, there was a significant difference in the change in arterial O_2_ saturation, with a larger change observed for the hypoxic exposure relative to the normoxic exposure (SpO_2_: 0.3% 95% CI: 0.2, 0.4, *p* < 0.0001, 0.36% vs. 0.1%) (Figure 2, Figure 3, Figure 4 and Figure 5).

## 4. Discussion

The purpose of the current study was to evaluate the responses of the ANS to low- and high-cognitive-load tasks performed under hypoxic and normoxic conditions. We hypothesized that (1) subjects would elicit a greater ANS response while performing high-cognitive-load tasks relative to low-cognitive-load tasks, and that (2) the ANS responses would be exacerbated under the hypoxic condition compared to the normoxic condition. The major findings of the study were (1) that performing higher-cognitive-load tasks elicited a negative ANS response reflected by higher HR and lower HRV; (2) that the heightened ANS activity observed occurred in both the hypoxic and normoxic conditions, but to a greater extent in the former condition; and (3) that performing lower-cognitive-load tasks, regardless of condition, appeared to decrease HR and increase HRV, possibly suggesting a period of ANS “recovery”.

Observing significant, differential responses of the ANS to varying levels of cognitive load under both hypoxic and normoxic conditions provides a novel contribution to the existing scientific literature. Under the hypoxic condition, the military aircrew exhibited heightened ANS activity while performing the MATB-II assessment, which utilized higher-order cognitive functions. Relative to the performance during the ADVT assessment, the ANS response to the MATB-II was significantly different and in the opposing direction. Aircrew elicited higher HR (2.4 bpm vs. −2.6 bpm, Δ 5.0 bpm, *p* < 0.0001) and reduced HRV indices (rMSSD: −0.4 ms vs. 1.8 ms, Δ −2.2 ms, *p* = 0.03 and SDNN: −13.6 ms vs. 0.7 ms, Δ −14.3 ms, *p* < 0.0001). This differential response is both interesting and unexpected. Physiologically, engaging in complex tasks increases cerebral O_2_ demand, requiring greater cerebral blood flow, a process regulated by the ANS [3,5,22]. Intuitively, we anticipated heightened ANS activity during the MATB-II assessment, which the current findings support. However, the reduced ANS activity found while aircrew performed the ADVT under a hypoxic condition was unexpected. Given the considerable and sustained drop in O_2_ saturation observed in the parent study at the onset of and throughout hypoxic exposure [11], it was assumed that performing any cognitive task, regardless of complexity, would elicit increased ANS activity. Instead, during the ADVT, following an initial 27 min exposure to hypoxia (epoch 1), aircrew elicited reductions in ANS activity, reflected by decreased HR and increased HRV. Interestingly, following each ADVT assessment and accompanying the one-minute rest, ANS activity subsequently increased during the MATB-II, most evidently for HR and SDNN values. This alternating pattern of reduced and heightened ANS activity observed during the ADVT and MATB-II assessments, respectively, may suggest that the ANS transiently “recovered” prior to aircrew performing the higher-order cognitive tasks of the MATB-II [23].

The posited “recovery” of the ANS observed in aircrew exposed to a low cognitive load under hypoxic conditions highlights two situations in which this response may improve or negligibly affect aircrew task performance. During active or training combat missions, aircrew execute tasks of varying complexity in fluctuating environmental conditions for prolonged periods of time [1,2,9,13]. Should the ANS response truly “recover” during the moments where aircrew perform low cognitive tasks, this may result in improved performance of subsequent cognitively demanding tasks. This is especially critical in the presence of hypoxia, even at mild levels, where aircrew spend a significant proportion of time while in flight [7,24,25]. Whether this same alternating pattern emerges at higher altitudes remains to be elucidated, yet it is an important avenue for future studies as, in flight, aircrew oscillate between varying levels of altitude.

Although aircrew perform both low- and high-cognitive-load tasks, the predictability of their occurrence and duration is incalculable. Therefore, the observed “recovery” period may not genuinely exist or significantly affect cognitive performance due to the design limitations of the parent study and the presence of influential confounding factors. The parent study, detailed elsewhere [11], occurred within a laboratory setting with zero risk of catastrophic outcomes and a controlled, predictable exposure to low and high cognitive loads. Thus, the design of the experiment did not adequately mimic the situations aircrew face during combat missions. Problematically, it is highly likely that the ANS is heightened in advance of flight operations [26,27,28,29]. The anticipation of potential danger, threat of death, and uncertainty trigger a flight-or-fight response, which is characterized by elevated heart rate (HR) and decreased heart rate variability (HRV). Subsequently, aircrew, while in flight, endure additional increases in ANS activity while performing cognitively demanding tasks of unpredictable duration. We speculate that the transient “recovery” observed in the current study may not appreciably alleviate the likely cumulative impact of pre-flight and cognitive load increases in ANS activity.

Under normoxic conditions, another differential response of the ANS to cognitive load was observed, albeit to a lesser degree. Compared to the ADVT, while performing the MATB-II, aircrew elicited significantly higher HR throughout epochs 2 through 4. (HR: 1.8 vs. −2.5 bpm, Δ 4.3 bpm, 95% CI: 1.2, 7.4, *p* = 0.002). While a reduction in HRV appeared to occur, paralleling the hypoxic condition, it did not reach statistical significance (rMSSD: −0.3 vs. 1.03 ms,Δ −1.3 ms, 95% CI: −3.5, 0.8, *p* = 0.39 and SDNN: Δ −2.0 ms, 95% CI: −0.11, 0.01, *p* = 0.61). This finding highlights a few important concepts. First, the similar response of the ANS to cognitive load under the normoxic exposure indicates that the difficulty of the cognitive tasks affects the ANS independent of mild hypoxia. While the observed difference might be deemed modest and possibly less clinically impactful, it emphasizes the importance of acknowledging the influence of cognitive load on ANS function. This becomes especially critical when considering the added effects of pre-flight increases in ANS activity and those arising from mild to severe hypoxic exposure.

Another unexpected, yet interesting, finding of the current study was observing that the metrics measuring ANS activity were impacted differently depending on the cognitive load and hypoxic/normoxic exposure. Specifically, HR, rMSSD, and SDNN were all significantly affected and to a greater extent during the MATB-II assessment under hypoxic conditions. For HRV, SDNN elicited the largest changes, which persisted across epochs 2 through 4, relative to rMSSD, which, although significant, changed minimally. Some research suggests that SDNN is more influenced by the sympathetic drive rather than vagal tone [30,31,32]. In the current study, the dual exposure to hypoxia and high cognitive load may have more strongly influenced sympathetic nervous system activity than vagal tone, reflected by higher SDNN values. In further support, other studies previously reported substantial decreases in SDNN consequent to physiological, cognitive, and psychological stressors [12,33,34]. Under normoxic conditions, however, HR was the only ANS metric that significantly differed between the MATB-II and ADVT assessments. Collectively, these observations may suggest that (1) the impacts of high cognitive load and hypoxia on ANS activity may be additive, supported by the larger magnitude and number of ANS metrics affected under these conditions, and (2) that monitoring ANS activity through multiple metrics, ideally within one device like the WFM, may be more accurate in tracking health-related outcomes during flight operations [10,11].

There are significant strengths to the current study. First, the design allowed us to compare the ANS response to two different levels of cognitive load for nearly two hours. While former studies evaluating ANS response and varying cognitive loads exist, many utilized short-duration task assessments, did not vary task complexity, and/or administered cognitive task assessments unrelated to a subjects’ occupation (e.g., the Stroop test). Second, our study additionally evaluated this relationship under hypoxic and normoxic conditions, environments to which aircrew are frequently exposed. Third, several ANS response metrics were measured using a single, non-invasive device (WFM) that possessed electrocardiographic capabilities that were strongly validated with the gold standard measure, electrocardiography (EKG). Fourth, the subjects served as their own controls, reducing the potential dissimilarities between the use of two separate samples. In addition to its strengths, the current study has limitations that warrant attention. First, while the alternating pattern of subjects performing low- and high-cognitive-load tasks identified the potential “recovery” of the ANS, we are unable to separate the possible residual influence of the ADVT and MATB-II tests on ANS function across the trial. Second, because the study design scheduled the ADVT test prior to the MATB-II test, the impact of the MATB-II test on ANS function may have been larger.

## 5. Conclusions

The associations between the performance of low and high cognitive loads and ANS function observed in the current study highlight a few important concepts related to the health and safety of military aircrew. First, under both mild hypoxic and normoxic conditions, environments in which aircrew spend much of their time during flight operations, performing higher-cognitive-load tasks elicited a significantly larger and more negative impact on ANS activity compared to lower cognitive loads. Subjects elicited higher HR and lower HRV, most notably SDNN, an indicator of sympathetic drive. Additionally, the ANS response was greater under mild hypoxia relative to normoxia. Lastly, the smaller ANS response to the lower-cognitive-load tasks under hypoxic conditions may suggest a “recovery” period; however, more research is needed.

### Future Directions

For future investigations, we strongly recommend designing studies measuring the ANS response during combat training missions to better estimate the impact on ANS function. During active or training combat missions, the duration and frequency of performing varying levels of cognitive loads is unpredictable. Moreover, ANS activity is likely heightened due to pre-flight activities and in-flight fight-or-flight response, which cannot be replicated in laboratory settings. This increased rigor in study design will enable researchers and military personnel to more accurately identify effective strategies to protect the health and safety of aircrew.

U.S. Army Disclaimer: The views, opinions, and/or findings contained in this presentation are those of the author(s) and should not be construed as an official Department of the Army position, policy, or decision, unless so designated by other official documentation. Citation of trade names in this presentation does not constitute an official Department of the Army endorsement or approval of the use of such commercial items.

## Figures and Tables

**Table 1:** Mean changes in the ANS response to high and low cognitive loads by conditions of Factor A.

	Hypoxic		Normoxic	
	MATB-II	ADVT	MATB-II	ADVT
	Mean Change (SD)	Mean Change (SD)	Mean Change (SD)	Mean Change (SD)
Epoch 2				
Heart rate (bpm)	1.8 (5.7)	−2.7 (5.0)	2.6 (3.96)	−3.3 (3.8)
rMSSD (ms)	−0.4 (2.4)	1.7 (4.9)	0.3 (0.2)	0.99 (5.2)
SDNN (ms)	−13.8 (15.7)	0.97 (5.6)	0.1 (1.6)	2.7 (5.7)
SpO_2_ (%)	−0.2 (0.1)	0.14 (0.01)	−0.2 (0.1)	0.02 (0.01)
Epoch 3				
Heart rate (bpm)	1.04 (8.7)	−2.0 (7.04)	−0.6 (8.8)	−1.9 (2.7)
rMSSD (ms)	−0.9 (3.8)	3.01 (6.7)	0.3 (4.2)	0.2 (2.9)
SDNN (ms)	−13.6 (15.1)	0.9 (6.7)	−0.30 (1.5)	0.7 (2.8)
SpO_2_ (%)	0.2 (0.03)	0.8 (0.2)	0.2 (0.03)	0.04 (0.02)
Epoch 4				
Heart rate (bpm)	4.4 (5.6)	−3.2 (7.0)	3.3 (5.2)	−2.3 (5.2)
rMSSD (ms)	0.03 (1.5)	0.5 (6.5)	−1.5 (2.3)	1.99 (4.2)
SDNN (ms)	−13.4 (14.1)	0.3 (6.7)	−0.7 (1.2)	1.8 (3.7)
SpO_2_ (%)	0.2 (0.00)	0.2 (0.02)	0.2 (0.00)	0.2 (0.1)
Epochs (2–4)				
Heart rate (bpm)	2.4 (6.9)	−2.6 (6.3)	1.8 (6.4)	−2.51 (3.9)
rMSSD (ms)	−0.4 (2.7)	1.8 (6.04)	−0.3 (2.8)	1.0 (4.2)
SDNN (ms)	−13.6 (14.7)	0.7 (6.3)	−0.3 (1.4)	1.7 (4.3)
SpO_2_ (%)	0.1 (0.3)	0.4 (0.3)	0.1 (0.2)	0.1 (0.10)

**Table 2:** Mean between-group differences in HR, rMSSD, SDNN, and SpO_2_ between ADVT and MATB-II by Factor A conditions.

	Hypoxia		Normoxia	
	MATB-II vs. ADVT		MATB-II vs. ADVT	
	Mean Difference (95% CI)	*p*-Value	Mean Difference (95% CI)	*p*-Value
Epoch 2				
Heart rate (bpm)	4.5 (−2.8, 11.8)	0.74	5.9 (−1.9, 13.6)	0.40
rMSSD (ms)	−2.1 (−7.1, 2.9)	0.99	−0.7 (−6.7, 4.5)	0.99
SDNN (ms)	−14.7 (−24.4, −5.0)	<0.0001	−2.6 (−12.6, 7.3)	0.99
SpO_2_ (%)	−0.5 (−1.2, 3.0)	0.82	−0.2 (−0.9, 0.6)	0.99
Epoch 3				
Heart rate (bpm)	3.0 (−4.3, 10.3)	0.99	1.3 (−6.6, 1.9)	0.99
rMSSD (ms)	−3.9 (−8.9, 1.1)	0.34	0.2 (−5.2, 5.6)	0.99
SDNN (ms)	−14.4 (−24.2, −4.6)	<0.0001	−0.9 (−11.0, 9.2)	0.99
SpO_2_ (%)	−0.4 (−1.2, 0.4)	0.92	−0.1 (−0.6, 0.9)	0.99
Epoch 4				
Heart rate (bpm)	7.6 (0.1, 15.1)	0.04	5.6 (−2.4, 13.6)	0.55
rMSSD (ms)	−0.5 (−5.7, 4.7)	0.99	3.5 (−1.9, 8.9)	0.69
SDNN (ms)	−13.6 (−23.6, −3.7)	0.0003	2.6 (−7.6, 12.8)	0.99
SpO_2_ (%)	−0.02 (−0.8, 0.8)	0.99	−0.02 (−0.8, 0.8)	0.99
Epoch 2–4				
Heart rate (bpm)	5.0 (2.1, 7.9)	<0.0001	4.3 (1.2, 7.4)	0.002
rMSSD (ms)	−2.2 (−4.2, −0.2)	0.03	−1.3 (−3.5, 0.8)	0.39
SDNN (ms)	−14.3 (−18.4, −10.1)	<0.0001	−2.0 (−6.3, 2.2)	0.61
SpO_2_ (%)	−0.3 (−0.4, −0.2)	<0.0001	−0.01 (−0.11, 0.01)	0.99

**Table 3:** Mean within-group differences in HR, rMSSD, SDNN, and SpO_2_ between Factor A conditions by cognitive load assessment.

	MATB-II		ADVT	
	Hypoxia vs. Normoxia		Hypoxia vs. Normoxia	
	Mean (95% CI)	*p*-Value	Mean (95% CI)	*p*-Value
Epoch 2				
Heart rate (bpm)	−0.8 (−8.3, 6.8)	0.99	0.6 (−6.9, 8.1)	0.99
rMSSD (ms)	−0.6 (−5.8, 4.6)	0.99	0.7 (−4.4, 5.9)	0.99
SDNN (ms)	−13.8 (−23.8, 3.9)	0.0002	−1.7 (−11.4, 7.9)	0.99
SpO_2_ (%)	−0.2 (−0.9, 0.6)	0.99	0.1 (−0.7, 0.9)	0.99
Epoch 3				
Heart rate (bpm)	1.7 (−5.9, 9.3)	0.99	−0.1 (−7.6, 7.4)	0.99
rMSSD (ms)	−1.3 (−6.5, 3.9)	0.99	2.9 (−2.3, 8.0)	0.88
SDNN (ms)	−13.3 (−23.5, −3.0)	0.001	0.2 (−9.5, 9.9)	0.99
SpO_2_ (%)	0.2 (−0.6, 0.9)	0.99	0.7 (−0.03, 1.5)	0.08
Epoch 4				
Heart rate (bpm)	1.1 (−6.6, 8.9)	0.99	−0.9 (−8.6, 6.9)	0.99
rMSSD (ms)	1.6 (−3.8, 6.9)	0.99	−1.5 (−6.8, 3.9)	0.99
SDNN (ms)	−12.6 (−22.9, −2.4)	0.003	−1.5 (−11.5, 8.4)	0.99
SpO_2_ (%)	−0.04 (−0.8, 0.7)	0.99	0.03 (−0.8, 0.7)	0.99
Epoch 2–4				
Heart rate (bpm)	0.6 (−2.4, 3.7)	0.95	−0.1 (−3.1, 2.9)	0.99
rMSSD (ms)	−0.1 (−2.2, 1.9)	0.99	0.7 (−1.3, 2.8)	0.79
SDNN (ms)	−13.3 (−17.5, −8.9)	<0.0001	−1.0 (−5.1, 3.1)	0.92
SpO_2_ (%)	−0.01 (−0.1, 0.1)	0.99	0.3 (0.2, 0.4)	<0.0001

## Data Availability

Data may be provided upon request.
